# Peri‐Implant Conditions During Supportive Periodontal Care: A Prospective Study

**DOI:** 10.1111/clr.70026

**Published:** 2025-08-28

**Authors:** Margherita Sforza, Pasquale Santamaria, Aliye Akcalı, Luigi Nibali

**Affiliations:** ^1^ University of Bologna – Alma Mater Studiorum Bologna Italy; ^2^ Periodontology Unit, Centre for Host Microbiome Interactions, Faculty of Dentistry, Oral and Craniofacial Sciences King's College London London UK; ^3^ Department of Periodontology, Faculty of Dentistry Dokuz Eylul University Izmir Türkiye

**Keywords:** maintenance, peri‐implantitis, prognosis

## Abstract

**Aim:**

To assess peri‐implant conditions in a cohort of patients previously treated for periodontitis undergoing supportive periodontal care (SPC).

**Materials and Methods:**

A prospective observational study was carried out on previously treated periodontitis patients followed for 5 years in SPC. Peri‐implant diagnosis at baseline and the end of the 5‐year period was assessed.

**Results:**

Two hundred patients were included in the study. A total of 88 implants were present at baseline in 31 of those patients. A total of 55 (62.5%) implants were diagnosed as having healthy peri‐implant tissues, while 22 (25%) were diagnosed with peri‐implant mucositis and 11 (12.5%) with peri‐implantitis. Five‐year data are available for 68 implants in 23 patients. None of these implants was lost during the 5 years follow‐up. An additional 20 implants were placed during the study period, resulting in a total of 88 implants reassessed at the last study follow‐up, with peri‐implant diagnosis almost unchanged compared with baseline. None of the studied patient and implant factors were associated with implant diagnosis in a multilevel model with logistic regression target distribution.

**Conclusions:**

A small percentage of implants in a population undergoing SPC were diagnosed with peri‐implantitis, and peri‐implant conditions were maintained almost unchanged during 5 years of SPC.

## 1 Introduction

Peri‐implant diseases and conditions pose significant challenges in the maintenance of dental implants and can lead to implant failure if not effectively managed. As the prevalence of implant therapy continues to rise, understanding the dynamics of peri‐implant health during supportive peri‐implant care (SPIC) becomes increasingly essential. Recent studies have highlighted that the management of peri‐implantitis and other related conditions is paramount for maintaining implant longevity and preventing complications (Cho‐Yan Lee et al. 2012; Ravidà et al. 2022).

A substantial body of evidence indicates that a history of periodontitis is a critical risk factor for the development of these conditions, as patients with previous periodontal disease often exhibit altered immune responses and increased susceptibility to peri‐implant infections (Astolfi et al. 2022; Roccuzzo et al. 2022). The interplay between periodontal health and implant success underscores the importance of rigorous monitoring and intervention strategies throughout the lifespan of dental implants.

SPIC plays a crucial role in monitoring and maintaining peri‐implant health. It involves a structured approach focusing on the ongoing assessment, maintenance, and treatment of peri‐implant tissues to prevent disease progression, addressing both biological and mechanical factors that may contribute to peri‐implant diseases (Heitz‐Mayfield et al. 2018).

The concept of SPIC has evolved, emphasizing the need for personalized care protocols that are responsive to the individual patient's risk factors, including oral hygiene practices, systemic health conditions, and an increase in microbial dental plaque scores (Outatzis et al. 2024).

Research has shown that regular SPIC can significantly enhance patient outcomes, reducing the incidence of complications associated with implant therapy (Costa et al. 2012). Effective SPIC not only involves professional mechanical plaque control but also emphasizes reinforcement of oral hygiene practices and self‐care. Long‐term studies have demonstrated the vital role of SPIC in preserving peri‐implant health. For instance, it has been reported that patients receiving regular SPIC exhibited a significantly lower incidence of peri‐implantitis compared to those who did not (Roos‐Jansåker et al. 2006). Furthermore, another study highlighted that the implementation of tailored SPIC protocols resulted in improved clinical parameters, including reduced probing depths and peri‐implant inflammation scores, thereby contributing to the longevity of implants (Rokn et al. 2017). These findings reinforce the necessity of a proactive approach in managing peri‐implant conditions, particularly for individuals at higher risk due to periodontitis history.

Despite the growing body of literature on peri‐implant conditions, there remains a lack of prospective studies that systematically evaluate the impact of SPIC on the clinical outcomes of patients with dental implants. By focusing on a cohort of patients receiving regular supportive peri‐implant care, this study will contribute to the understanding of how these interventions influence peri‐implant health and inform future clinical practices. Therefore, this study aimed to assess peri‐implant conditions in a cohort of patients with periodontitis undergoing supportive periodontal care (SPC) in a private practice setting in the United Kingdom.

## 2 Materials and Methods

### 2.1 Patient Population

The materials and methods and results related to teeth in this cohort have been reported elsewhere (Hasan et al. 2024). Two hundred consecutive patients enrolled in a SPC programme were recruited from author LN's patient list in three private periodontal practices in London and Bishop's Stortford, United Kingdom. All patients had been referred to author LN for periodontal care. Ethics approval for the analysis was sought from The London and City Ethics Committee, which gave permission for the study to be carried out as a service evaluation (reference 14 LO 0629). Each patient gave written consent to take part in the study. The study was registered on clinicaltrials.gov (NCT02091258). Patient visits took place from August 2014 to June 2021. The following inclusion criteria were considered for patient recruitment: (i) diagnosis of chronic periodontitis with interproximal attachment loss ≥ 3 mm in at least 2 non‐adjacent teeth (Tonetti, et al. 2005); (ii) at least 2 sites with ≥ 5 mm probing pocket depths (PPDs) and radiographic evidence of bone loss ≥ 20% of root length at the first visit; (iii) treated by author LN with non‐surgical periodontal treatment (NSPT) with or without subsequent periodontal surgeries; (iv) willing to give written informed consent for study participation; (v) willing to undergo SPC as per standard of care for at least 5 years.

Exclusion criteria were (i) serious medical history that prevented patients from undergoing dental treatment; (ii) conditions requiring prophylactic antibiotic coverage prior to invasive dental procedures; (iii) current alcohol or drug abuse; (iv) self‐reported pregnancy or lactation; (v) other severe acute or chronic medical or psychiatric conditions or laboratory abnormalities that may compromise trial participation and/or interpretation of trial results.

STROBE guidelines were followed for reporting the study (von Elm et al. 2008).

### 2.2 Pre‐Study Periodontal/Peri‐Implant Therapy

Initial periodontal therapy, prior to study baseline, consisted of case presentation and patient motivation, oral hygiene instruction (OHI) and non‐surgical supra‐ and sub‐gingival professional mechanical plaque removal (PMPR) followed by, as required, periodontal surgery including access flap, regenerative and mucogingival surgery, and endodontic and prosthetic treatment if necessary. Some patients received adjunctive therapy including systemic or local antibiotics (Sanz et al. 2020). Teeth which were considered irrational to treat according to the initial treatment plan were extracted during initial periodontal therapy. Implants were treated according to clinical judgment, as part of periodontal therapy, mostly with oral hygiene instructions and supra‐ and sub‐marginal instrumentation with a combination of ultrasonic and hand instruments. Patients were then assessed 3–6 months later and, if periodontal conditions were considered stable, entered SPC.

### 2.3 Clinical Examinations

Following written consent, at baseline, self‐reported patient medical and smoking history was checked. The following periodontal measurements were taken by author LN at six sites/tooth: full mouth plaque scores (FMPS), full mouth probing pocket depth (PPD), recession (REC) of the gingival margin from the cemento‐enamel junction (CEJ), full mouth bleeding on probing (FMBS) (Ainamo and Bay 1975), tooth mobility (Laster et al. 1975) and furcation involvement (Hamp et al. 1975; Tarnow and Fletcher 1984). Clinical attachment level (CAL) was calculated as PPD + REC. Implants and relative restorations were probed in the same fashion, considering the crown margin as a reference instead of the CEJ. There was no instance where probing could not be assigned to the implant due to overhanging restorations. Dental radiographs of each patient had been obtained as necessary for diagnosis and treatment planning purposes at this visit. Disease risk was calculated at baseline using the Periodontal Risk Assessment (PRA) (Lang and Tonetti 2003), modified not to include genetic factors (Persson 2003).

### 2.4 Assignment of Implant Diagnosis

The peri‐implant diagnosis was assigned retrospectively by the calibrated examiner (author MS) using the guidelines of the 2018 classification (Araujo and Lindhe 2018; Berglundh et al. 2018; Heitz‐Mayfield and Salvi 2018; Schwarz et al. 2018). In the absence of previous examination data, the diagnosis of peri‐implantitis was based on the combination of:

- Presence of bleeding and/or suppuration on gentle probing.
- Probing depths of ≥ 6 mm.
- Bone levels ≥ 3 mm apical of the most coronal portion of the intraosseous part of the implant.

### 2.5 SPIC Protocol

SPIC was carried out as part of a SPC protocol. SPC followed an individualized interval of 3–12 months and consisted of medical and dental history updates, pocket charts, oral hygiene re‐instructions and motivation, and supra‐ and sub‐gingival debridement (under local anaesthesia when necessary). Study visits, including full periodontal charting as described above, were carried out every 12 months. SPC recall intervals were individualised based on PRA combined with patient preferences. SPC visits were carried out by author LN. If deterioration in periodontal parameters was detected, further treatment (including periodontal surgeries, extractions, or endodontic therapy) was carried out. The same approach was taken for the maintenance of dental implants. When progression was detected, further treatment, usually consisting of subgingival debridement under local anaesthesia, was carried out.

### 2.6 Radiographic Analyses

Periapical radiographs from all patients included in the study were screened, entered in a dedicated database, transferred into a dedicated software system (Xposeit version 3.01, Torben Jørgensen, Lystrup, Denmark) and analyzed by one designated examiner (author A.A.) at all measurable sites (mesial and distal) (Nibali et al. 2011).

### 2.7 Examiner Calibration

A calibrated examiner (author L.N.) carried out all study visits. Results of calibration have previously been reported (Hasan et al. 2024). Peri‐implant diagnosis was assigned prospectively clinically by author LN. For standardization purposes, all implants diagnosis at baseline were then retrospectively assigned by a single calibrated examiner (author M.S.). Reproducibility of implant diagnosis was carried out by assigning peri‐implant diagnosis twice 1 week apart to 25 non‐study cases based on clinical and radiographic data by the examiner (author M.S.), resulting in 96% agreement between first and second diagnoses. In cases when radiographic data at baseline were not retrievable, the original clinical diagnosis was used.

### 2.8 Sample Size Calculation and Statistical Analysis

The sample size for this study was calculated based on smoking as a risk factor for tooth loss (Hasan et al. 2024). Therefore, the study was not powered for any implant‐related outcome. The secondary analyses described here aimed at assessing the diagnosis of peri‐implant conditions at baseline and up to 5 years follow‐up and at assessing factors associated with peri‐implant diagnosis. Two‐sided tests were used for all analyses, and the level of statistical significance was set at 5%. All statistical analysis procedures were performed with IBM SPSS 23.0.

A multilevel model with logistic regression target distribution was used to account for the dependency of outcomes on multiple teeth in individual patients (Cho and Kim 2015). Predictor variables, including age, sex, smoking, presence of PPDs > 4 mm at baseline, stage, grade, years since implantation, and bridge abutment, were included in the model as fixed effects. Due to collinearity between stage and presence of PPDs > 4 mm at baseline, two models were tested, containing either of these two parameters.

## 3 Results

### 3.1 Baseline Characteristics

The ‘baseline’ of this study corresponds with when patients signed the consent form and were officially enrolled in the study. As previously reported (Hasan et al. 2024), most patients were diagnosed as stage III (87%), with a lower proportion of stage IV periodontitis (13%), and most patients were assigned a grade B (61%), followed by grade C (39%) (Tonetti et al. 2018). The extent of periodontitis cases was equally distributed (50% localized and 50% generalized).

### 3.2 Patient Flow

**FIGURE 1:**
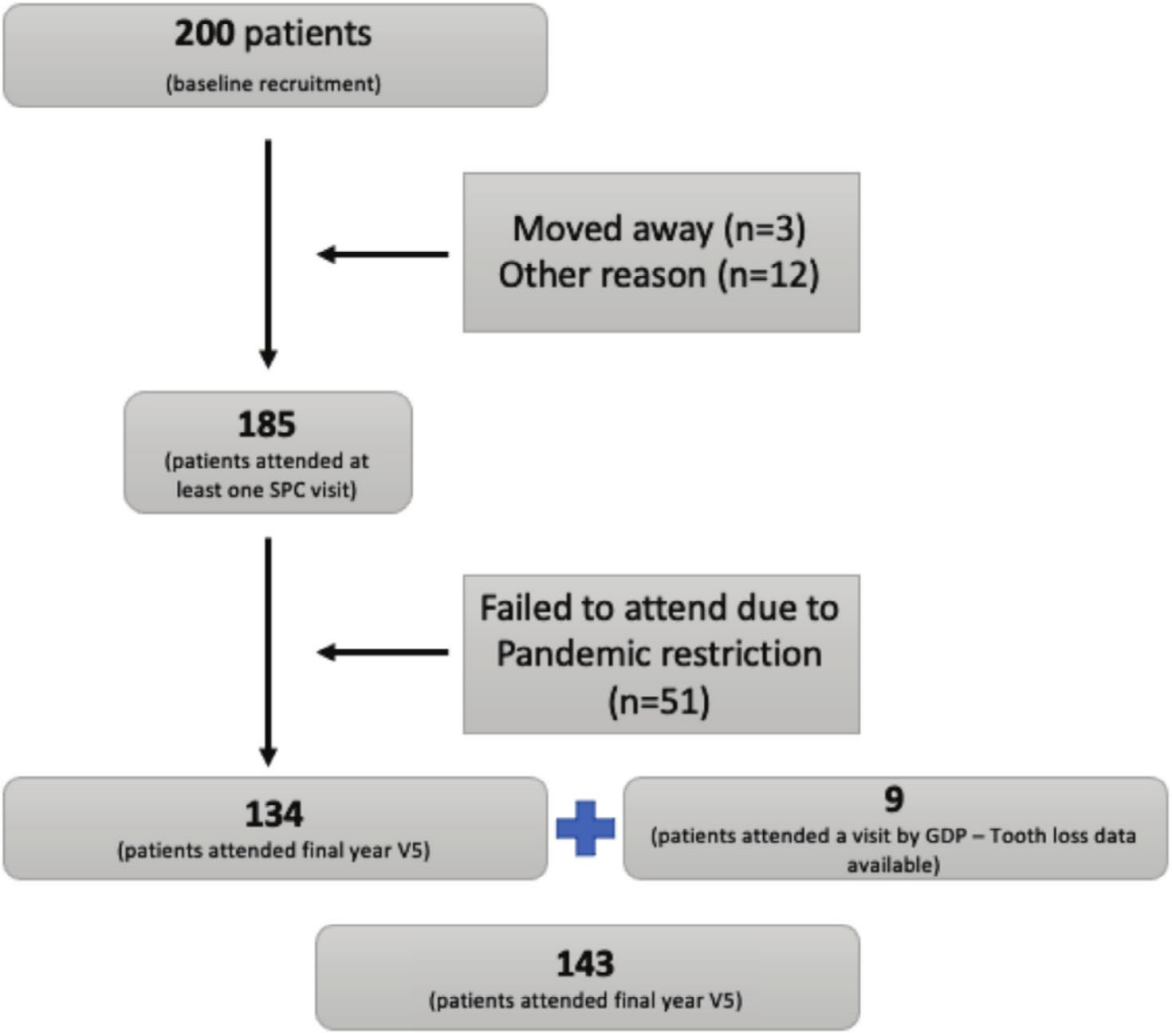
Flowchart of the participants of the study. Taken from Hasan et al. (2024).

A decreasing number of patients attended follow‐up study visits, from baseline (*n* = 200) to year‐5 (*n* = 143) (Figure 1). This was discussed in the original publication (Hasan et al. 2024) and may be partially related to financial issues, as well as to the Covid pandemic. Out of the 143 patients with 5‐year data, the majority (69.9%) was compliant with all follow‐up visits.

### 3.3 Baseline Implant Diagnosis

Table 1 describes the features of the implants present at baseline in all patients included in the study. A total of 88 implants were present at baseline across all 200 patients. Thirty‐one patients had at least one implant at baseline, while 169 patients did not have any; although 4 of these went on to receive implants during the study period.

**TABLE 1:** Baseline implant characteristics.

Time since implantation (years)		5.55 ± 4.07
Location	Maxilla	56 (63.6%)
	Mandible	32 (36.4%)
Region	Anterior (incisor‐canine)	27 (30.7%)
	Premolar	25 (28.4%)
	Posterior (molar)	36 (40.9%)
Peri‐implant disease diagnosis	Peri‐implant health	55 (63.5%)
	Peri‐implant mucositis	22 (25.0%)
	Peri‐implantitis	11 (12.5%)
Average worse PPD around implant (mm)		4.00 ± 1.07
Average worse CAL around implant (mm)		4.38 ± 1.23
BOP positive (at least 1 site)		33 (37.5%)

Out of the 88 implants present at baseline, 55 (62.5%) were diagnosed as having healthy peri‐implant tissues, while 22 (25%) were diagnosed with peri‐implant mucositis and 11 (12.5%) with peri‐implantitis. Average PPD and CAL were 4.0 ± 1.1 mm and 4.4 ± 1.2 mm respectively, while 37.5% of peri‐implant tissues exhibited at least 1 site which bled on probing. Table 2 shows associations between periodontal diagnosis at the start of SPC, defined as stage, grade, ‘stability’ (Sanz et al. 2020), ‘controlled periodontitis’ (Feres et al. 2020) and absence of PPD > 4 mm, and baseline peri‐implant diagnosis, showing a non‐statistically significant tendency to worse peri‐implant diagnosis for patients with stage IV (compared with III). However, this analysis is not adjusted for the multilevel nature of the data.

**TABLE 2:** Associations between patient periodontal conditions and implant diagnosis at baseline and 5 years (not adjusted for multilevel nature of data).

Patient diagnosis at baseline		Baseline peri‐implant diagnosis			*p* =	5‐year peri‐implant diagnosis			*p* =
		Health	Peri‐implant mucositis	Peri‐implantitis		Health	Peri‐implant mucositis	Peri‐implantitis	
Stage	III	39 (69.6%)	13 (23.2%)	4 (7.1%)	0.083	34 (66.7%)	14 (27.5%)	3 (5.9%)	0.042
	IV	16 (50%)	9 (28.1%)	7 (21.9%)		21 (56.8%)	7 (18.9%)	9 (24.3%)	
Grade	B	41 (68.3%)	12 (20%)	7 (11.7%)	0.221	37 (68.5%)	11 (20.4%)	6 (11.1%)	0.336
	C	14 (50%)	10 (37.5%)	4 (14.3%)		18 (52.9%)	10 (29.4%)	6 (17.6%)	
‘Stable periodontitis’ (Sanz et al. 2020)	Yes	6 (85.7%)	1 (14.3%)	0	0.376	8 (88.9%)	1 (11.1%)	0	0.205
	No	49 (60.5%)	21 (25.9%)	11 (13.6%)		47 (59.5%)	20 (25.3%)	12 (15.2%)	
‘Controlled periodontitis’ (Feres et al. 2020)	Yes	42 (66.7%)	13 (20.6%)	8 (12.7%)	0.315	39 (65%)	13 (21.7%)	8 (13.3%)	0.747
	No	13 (52%)	9 (36%)	3 (12%)		16 (57.1%)	8 (28.6%)	4 (14.3%)	
Presence of PPD > 4 mm on any teeth	Yes	34 (60.7%)	14 (25%)	8 (14.3%)	0.790	30 (51.7%)	19 (32.8%)	9 (15.5%)	0.010
	No	21 (65.6%)	8 (25%)	3 (9.4%)		25 (83.3%)	2 (6.7%)	3 (10%)	

### 3.4 Implant Diagnosis at 5 Years

**FIGURE 2:**
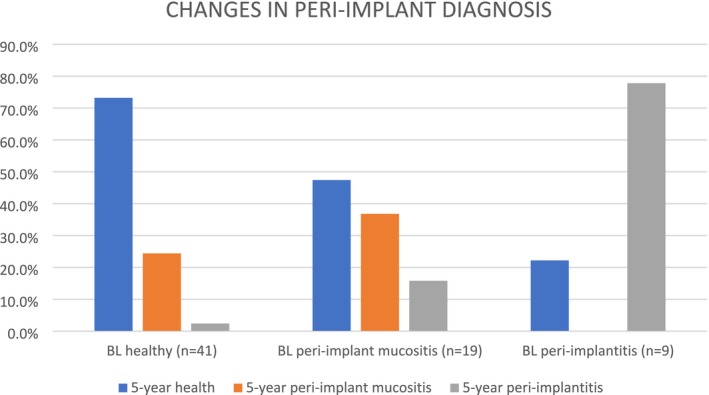
Comparison of baseline (BL) and 5‐year diagnoses (implants divided into healthy, peri‐implant mucositis and peri‐implantitis).

From the initial 31 included patients, eight (accounting for 20 implants) were lost to follow‐up before the 5‐year visit due to lack of contact or difficulties due to the COVID‐19 lockdowns. Therefore, 5‐year data are available for 68 implants in 23 patients. None of the implants were lost during the 5 years' follow‐up. An additional 20 implants were placed during the study period, resulting in a total of 88 implants reassessed at the last study follow‐up. Patients received periodontal supportive care during the 5 years, including supra‐ and sub‐marginal instrumentation of implants/implant crowns. Patients attended an average of 9.6 ± 10.5 visits with the periodontist and 10.5 ± 5.6 with the hygienist throughout the 5 years, and only one reconstructive/regenerative surgical procedure was carried out on 1 implant (with peri‐implantitis diagnosis) during this period. Figure 2 shows the relationships between baseline and 5‐year diagnoses. Implant diagnosis mostly remained the same during the 5‐year follow‐up, with a few implants going from healthy at baseline to peri‐implant mucositis and vice versa and only 2 implants going from peri‐implantitis at baseline to healthy diagnosis at 5 years. Average PPD and CAL were 4.0 ± 1.4 mm and 4.3 ± 1.5 mm, respectively, while 41.3% of peri‐implant tissues exhibited at least 1 site that bled on probing. Fifty‐five (62.5%) were diagnosed as having healthy peri‐implant tissues, while 21 (23.9%) were diagnosed with peri‐implant mucositis and 12 (13.6%) with peri‐implantitis. Table 2 shows associations between periodontal diagnosis at the start of SPC (stage, grade, ‘stability’, ‘controlled periodontitis’ and absence of PPD > 4 mm) and 5‐year peri‐implant diagnosis. Associations between both staging and presence of PPD > 4 mm on any teeth and 5‐year implant diagnosis were found at univariate analysis. In the two multilevel models with logistic regression target distribution, only the presence of PPD > 4 mm at baseline predicted implant diagnosis at 5 years (*p* = 0.015 for diagnosis of peri‐implant health vs. peri‐implant diseases) (Table 3).

**TABLE 3:** Results of multilevel model with logistic regression, with peri‐implant diagnosis at 5 years as target. Two models are presented, the first including staging and the second presence of PPD > 4 mm at baseline (instead of stage) for both peri‐implant health (vs. peri‐implant disease) and peri‐implantitis (vs. health/peri‐implant mucositis).

	Peri‐implant health (vs. peri‐implant disease)		Peri‐implantitis (vs. health/peri‐implant mucositis)	
	Model 1	Model 2	Model 1	Model 2
	*p* [OR (95% CI)]			
Age	0.455 [1.02 (0.96–1.09)]	0.282 [0.61 (0.95, 1.08)]	0.561 [0.96 (0.85, 1.09)]	0.664 [0.97 (0.87, 1.09)]
Gender (ref. female)	0.255 [2.19 (0.56, 8.49)]	0.264 [1.94 (0.60–6.29)]	0.524 [0.39 (0.02, 7.05)]	0.180 [0.20 (0.02, 2.13)]
Smoking (ref. no smoking)	0.535 [2.45 (0.14, 42.47)]	0.416 [3.06 (0.20, 46.33)]	0.971 [1.07 (0.02, 50.35)]	0.852 [1.40 (0.04, 51.76)]
Stage (ref. III)	0.836 [0.87 (0.22, 3.43)]	—	0.329 [3.64 (0.27, 49.79)]	—
Implant age	0.261 [1.07 (0.95, 1.22)]	0.176 [1.09 (0.28–2.43)]	0.622 [0.94 (0.73, 1.20)]	0.757 [0.97 (0.77, 1.20)]
Bridge abutment (ref. no abutment)	0.804 [0.87 (0.28, 2.64)]	0.725 [0.82 (−1.28, 0.89)]	0.577 [1.75 (0.24, 12.64)]	0.438 [2.04 (0.33, 12.69)]
Presence of PPD > 4 mm at baseline (ref. no PPDs > 4 mm)	—	0.015 [0.20 (0.05, 0.72)]	—	0.795 [1.31 (0.17, 10.25)]

## 4 Discussion

To the best of our knowledge, this is the first 5‐year prospective study to assess the efficacy of SPIC for long‐term implant maintenance in patients with a history of periodontitis. This investigation reported that supportive periodontal care was effective in maintaining implant diagnosis mostly unchanged during the 5‐year follow‐up. The long‐term stability of peri‐implant health is crucial for the success and functionality of dental implants. Achieving and maintaining this stability requires a combination of biological, mechanical, and behavioral factors. Although the main cause of peri‐implant disease is the accumulation of a dysbiotic plaque biofilm, important risk factors/indicators have been identified, including a history of severe periodontitis, poor plaque control, and no regular supportive peri‐implant care (SPIC) following implant therapy (Herrera et al. 2023).

Less than 15% of implants in the present sample were diagnosed with peri‐implantitis at the start of the maintenance period. These findings are in line with a prior study that found that, in patients receiving maintenance therapy, the incidence of peri‐implantitis was 18%; whereas it was higher in those not receiving professional care (44%) (Costa et al. 2012). In the present study, few implants went from healthy at the start of SPIC to peri‐implant mucositis and vice versa; no implants were lost, and only two implants went from peri‐implantitis at baseline to a healthy diagnosis at 5 years. This result is in line with a previous study that reported how, in the medium‐long term, peri‐implantitis treatment combined with consistent supportive care led to high patient and implant‐level survival. In particular, favorable outcomes were noted, with most patients exhibiting stable peri‐implant bone levels and clinical improvements (Roccuzzo et al. 2018). On the other hand, patients who do not adhere to recommended maintenance therapy frequently require more peri‐implantitis re‐treatment over a 10‐year period in contrast to those who attended follow‐up appointments (Roccuzzo et al. 2012). It is commonly accepted that the recurrence of peri‐implantitis at the patient level appears to be higher compared to the recurrence of periodontitis, with a high percentage of secondary peri‐implantitis after 3–5 years from the treatment (Stiesch et al. 2023). In general, this study demonstrated that regular SPIC after treatment of peri‐implantitis was an effective measure in preventing a recurrence of peri‐implantitis.

Interestingly, only the presence of a periodontal pocket deeper than 4 mm at the start of SPIC predicted implant diagnosis at 5 years, as cases with residual PPDs > 4 mm at the start of SPC had a higher chance of having peri‐implant diseases (mucositis or peri‐implantitis) at 5 years. Several studies have shown that deep periodontal pockets are a strong indicator of peri‐implant disease progression. In fact, periodontal pockets can facilitate the accumulation of plaque biofilm, which may be responsible for the onset and progression of peri‐implant diseases in adjacent implants. In contrast with previous literature, this study failed to correlate the peri‐implant status to well‐known risk factors such as BOP, poor plaque control, prosthetic factors, limited access to oral hygiene (OH), peri‐implant keratinized mucosa, and systemic factors such as smoking and diabetes. However, further research is required to clarify the true roles of each factor for peri‐implant diseases onset and/or progression, as, for example, keratinized mucosa was not consistently measured in the current sample (Schwarz et al. 2018).

This study's strength lies primarily in its targeting of a specific group of patients with periodontitis under SPIC and its evaluation of changes over a valuable follow‐up period. The significance of SPIC in maintaining peri‐implant health is demonstrated by the observation that peri‐implant conditions stayed essentially constant over a five‐year period. Furthermore, the lack of appreciable changes in peri‐implant diagnoses from baseline challenges the belief that patients with periodontitis are inherently likely to have disease progression once peri‐implant diseases are diagnosed. Despite its strengths, this study has several limitations. The relatively small sample size of patients and implants followed over 5 years, especially for patients with peri‐implantitis, along with the lack of sample size calculation for the implant outcomes, limit the generalizability of the findings. Another limitation of this study is that all patients were treated in a private clinical setting by a single operator. While this consistency minimizes variability in surgical and maintenance techniques, it may also limit the generalizability of the findings to other clinical environments. In private practices, patients often receive personalized care and may have higher levels of compliance with supportive periodontal care (SPC) protocols due to the closer patient‐provider relationship. This might not reflect outcomes in public healthcare systems or in practices with multiple operators, where variability in care delivery and patient adherence might lead to different peri‐implant health outcomes. Future research should consider multicenter studies involving diverse clinical settings, including public healthcare systems and clinics with multiple operators. This would provide a more comprehensive understanding of how treatment variability and practice environments influence long‐term peri‐implant health.

To conclude, these findings highlight the significance of SPC and more specifically SPIC in preserving the health of the peri‐implant in patients who have a history of periodontitis. The stability of peri‐implant conditions over a five‐year period confirms the importance of routine maintenance in halting the progression of the disease. While more research is necessary to improve SPIC protocols and investigate individual risk factors for peri‐implant diseases, these findings reinforce the need for clinicians to continue emphasizing patient compliance and routine monitoring.

## Author Contributions

**Margherita Sforza:** writing – original draft, investigation, writing – review and editing. **Pasquale Santamaria:** formal analysis, writing – original draft, investigation. **Aliye Akcalı:** formal analysis, writing – original draft, investigation, writing – review and editing. **Luigi Nibali:** conceptualization, data curation, formal analysis, visualization, writing – original draft, methodology, investigation, supervision, project administration, writing – review and editing, validation, resources.

## Conflicts of Interest

The authors declare no conflicts of interest.

## Data Availability

The data that support the findings of this study are available on request from the corresponding author. The data are not publicly available due to privacy or ethical restrictions.
